# Role of Transmembrane 4 L Six Family 1 in the Development and Progression of Cancer

**DOI:** 10.3389/fmolb.2020.00202

**Published:** 2020-08-18

**Authors:** Fangmei Fu, Xudong Yang, Minying Zheng, Qi Zhao, Kexin Zhang, Zugui Li, Hao Zhang, Shiwu Zhang

**Affiliations:** ^1^Graduate School, Tianjin University of Traditional Chinese Medicine, Tianjin, China; ^2^Tianjin Rehabilitation Center, Tianjin, China; ^3^Department of Pathology, Tianjin Union Medical Center, Tianjin, China; ^4^Graduate School, Tianjin Medical University, Tianjin, China; ^5^Nankai University School of Medicine, Nankai University, Tianjin, China

**Keywords:** TM4SF1, cancer, signaling pathway, migration, progression

## Abstract

Transmembrane 4 L six family 1 (TM4SF1) is a protein with four transmembrane domains that belongs to the transmembrane 4 L six family members (TM4SFs). Structurally, TM4SF1 consists of four transmembrane domains (TM1–4), N- and C-terminal intracellular domains, two extracellular domains, a smaller domain between TM1 and TM2, and a larger domain between TM3 and TM4. Within the cell, TM4SF1 is located at the cell surface where it transmits extracellular signals into the cytoplasm. TM4SF1 interacts with tetraspanins, integrin, receptor tyrosine kinases, and other proteins to form tetraspanin-enriched microdomains. This interaction affects the pro-migratory activity of the cells, and thus it plays important roles in the development and progression of cancer. TM4SF1 has been shown to be overexpressed in many malignant tumors, including gliomas; malignant melanomas; and liver, prostate, breast, pancreatic, bladder, colon, lung, gastric, ovarian, and thyroid cancers. TM4SF1 promotes the migration and invasion of cancer cells by inducing epithelial-mesenchymal transition, self-renewal ability, tumor angiogenesis, invadopodia formation, and regulating the related signaling pathway. TM4SF1 is an independent prognostic indicator and biomarker in several cancers. It also promotes drug resistance, which is a major cause of therapeutic failure. These characteristics make TM4SF1 an attractive target for antibody-based immunotherapy. Here, we review the many functions of TM4SF1 in malignant tumors, with the aim to understand the interaction between its expression and the biological behaviors of cancer and to supply a basis for exploring new therapeutic targets.

## Introduction

Transmitting signals between the extracellular and intracellular microenvironments is a fundamental function of the plasma membrane. During signal transduction, numerous proteins functionally associate with each other (Yanez-Mo et al., 2009). The tetraspanin superfamily, which currently consists of 33 proteins, including CD151, CD63, CD81, CD82, and CD9, is a group of transmembrane proteins located in the plasma membrane that are comprised 200–350 amino acids with molecular masses of 20–30 kDa (Yanez-Mo et al., 2009; Lee, 2015). Tetraspanin family was first recognized in 1990 (Hemler, 2003). TM4SF family members (TM4SFs) is a branch of the tetraspanin superfamily, which include TM4SF1, TM4SF4 (IL-TIMP), TM4SF5 (L6H), TM4SF18 (L6D), TM4SF19, and TM4SF20 (Zhang et al., 2001). TM4SF and other tetraspanin superfamily members share a basic homoplastic structure consisting of four transmembrane domains (TM1–4), N- and C-terminal intracellular domains, two extracellular loops, a smaller domain between TM1 and TM2, and a larger domain between TM3 and TM4. The slightly shorter loop in the extracellular domains is called extracellular loop 1 (EC1). The longer loop in the extracellular domains is called extracellular loop 2 (EC2), and it contains a three-α-loop (A,B, and E) and a variable region (Wright et al., 2000). Six or more cysteines are contained in the region of EC2, and these residues are important for TM4SFs binding with integrin. Almost all tetraspanins contain palmitoylated cysteine near the membrane. All tetraspanins include a highly conserved Cys-Cys-Gly (CCG) motif located behind the B helix of the EC2 loop (Detchokul et al., 2014). However, TM4SFs has several differences from other tetraspanin superfamily members. First, an LGC motif is present in the TM2 domain of the tetraspanin superfamily, but is absent in TM4SFs. Second, the major extracellular domain of the tetraspanins has four extraordinarily well-conserved cysteine residues in three motifs: CCG, Phe-X-Ser-Cys, and Glu-Gly-Cys. However, the same region of the TM4SFs has no conserved cysteine residues; instead, TM4SFs has two cysteine residues in other domains that are not present in tetraspanins. Third, tetraspanins have conserved charged or polar amino acid residues in the TM1 domain and glutamine residues/glutamic acid in the TM3 and TM4 domains. In TM4SFs, there are no conserved charged/polar residues checked, instead there are only two glutamine residues/glutamic acids in the TM4 domain. Lastly, there are 23 amino acids between the TM2 and TM3 domains in TM4SFs, but only five amino acids in other tetraspanins (Wright et al., 2000).

Despite their differences, TM4SFs and the tetraspanin superfamily have analogous biological functions. Both can form tetraspanin-enriched microdomains (TEMs or TERMs) through their interaction with other proteins, including integrins, receptor tyrosine kinases, immunoproteins, lipid kinases, and PDZ-domain-containing proteins (Lekishvili et al., 2008). TEMs play crucial roles in modulating various cellular functions, such as cell motility, adhesion, propagation, migration, and invasion through signaling pathways including activation of PI3-kinase and Cdc42 (Lee et al., 2011; Detchokul et al., 2014). On the cell surface, tetraspanins exist as dimers, e.g., CD9-CD9 and CD151-CD81, suggesting that the dimer might be the basic unit of TEM (Kitadokoro et al., 2002). Homomorphic dimers are considered to be the core unit of complexes both between tetraspanins and with other partners (Kovalenko et al., 2004). Integrin is the most important tetraspanin-interacting partner (Berditchevski, 2001), as these interactions are crucial for integrin-mediated cell adhesion to the extracellular matrix (ECM), which is related to the development of cancer. CD151 was the first tetraspanin associated with cancer development and progression (Sadej et al., 2014; Lee, 2015). CD151 is clustered at the cell membrane in specialized multimeric aggregates and can organize distribution and function of interacting laminin-binding integrins and matrix metalloproteinases (MMPs) in cancer cell migration and invasion (Sadej et al., 2014). The CD151–integrin α3ß1 complex located at the invadopodia and could regulate generation of MMPs through the PI3K pathway in many invasive carcinoma cells (Sugiura and Berditchevski, 1999). CD151/integrin α3/α6 complex formed a functional complex with c-Met in human salivary (Klosek et al., 2005; Sadej et al., 2014). Other tetraspanins, such as CD9, CD53, CD81, and CD82, regulate cell signaling, motility, tumor cell metastasis and can act as linker molecules to recruit protein kinase C (PKC) into proximity with integrin ß1 (Zhang et al., 2001).

Here, we review the diverse functions of TM4SF1 in numerous malignancies, with an aim to fully understand the interaction between the expression of this transmembrane protein and the biological behaviors of cancer and to provide a basis for exploring new therapeutic targets.

## Structure and Function of TM4SF1

**FIGURE 1:**
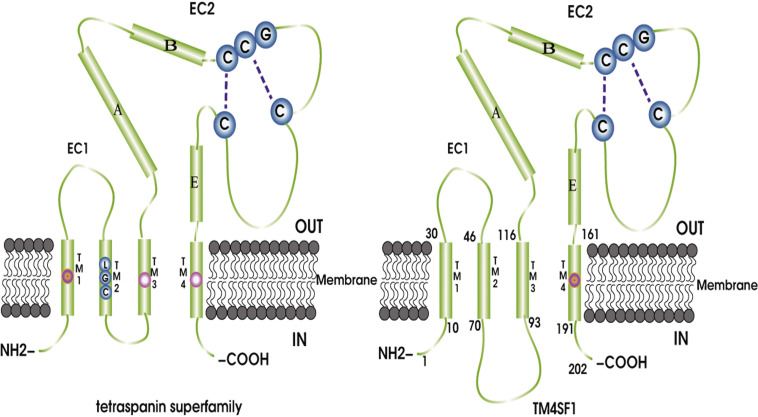
TM4SF and the tetraspanin superfamily share a basic homoplastic structure consisting of four transmembrane domains (TM1–4), N- and C-terminal intracellular domains, two extracellular domains, a smaller domain between TM1 and TM2, and a larger domain between TM3 and TM4. The slightly shorter loop in the extracellular domain is called SDE/SEL or EC1, and the longer loop in the extracellular domain is named LED/LEL or EC2 and contains a three-α-loop (A,B, and E) and a changing domain. Different regions, such as the LGC motif in the TM2 domain, exist in the tetraspanin superfamily, but are absent from TM4SF. In addition, tetraspanins have conserved charged or polar amino acid residues in the TM1 domain and glutamine residues/glutamic acid in the TM3 and TM4 domains. However, there are no conserved charged/polar residues in TM4SF; instead, there are two glutamine residues/glutamic acid residues in the TM4 domain. Finally, there are 23 amino acids between the TM2 and TM3 domains in TM4SF, but only five amino acids in the tetraspanins.

Transmembrane 4 L six family 1 (also known as L6 antigen, L6-Ag, TAL6, and L6) was initially identified in 1986 (Hellstrom et al., 1986b) and was a highly expressed surface protein of human lung, breast, colon, and ovarian carcinomas, which could be used as a cancer-specific antigen with the mouse monoclonal antibody L6 (mAb-L6) (Hellstrom et al., 1986a; Garkavij et al., 1995). Molecular cloning of TM4SF1 showed it to be a surface protein with four transmembrane domains, and initial analyses of TM4SF1 sequence suggested that the protein was a member of the tetraspanin (Hellstrom et al., 1986b; Marken et al., 1992). The TM4SF1 gene is located on human chromosome 3 at 3q21-q25 (Virtaneva et al., 1994). TM4SF1 was first identified as a tetraspanin superfamily member. It is a 202 amino acid protein with a molecular mass of approximately 22 kDa (Marken et al., 1992; Wright and Tomlinson, 1994) that contains four transmembrane domains (TM1–4), intracellular domains at the N- and C-termini, two extracellular domains, a small domain between TM1 and TM2, and a large domain between TM3 and TM4 (Figure 1). There are 23 amino acid residues between the TM2 and TM3 domains. The slightly shorter loop in the extracellular domain is called EC1. The longer loop in the extracellular domain, which is named EC2, contains three α loops (A,B, and E) and a variable region, which is located behind the B helix (Wright et al., 2000). The cytoplasmic portion of TM4SF1 in the C-terminus includes a non-traditional PDZ domain binding motif (X-TyrX-Cys) that may interact with syntenin-2 (SITAC), a PDZ domain-containing protein (Borrell-Page’s et al., 2000), and bind to syntenin-1, which then bridges the TM4SF1–syntenin-2 complex (Koroll et al., 2001). This interaction might be critical for the biological function of TM4SF1 because syntenin-1 is involved in cancer cell proliferation and migration (Sarkar, 2004). TM4SF1, TM4SF4, and TM4SF5 share 40–50% overall sequence identity and their C-terminal sequences differ substantially (Cheong et al., 2017).

Transmembrane 4 L six family 1 was detected at low or moderate levels in various normal human and mouse tissues, including the endothelium, skin, lung, and germ cells (Edwards et al., 1995; Tascou et al., 2001), In contrast, TM4SF1 has been shown to be highly expressed in the cytomembrane of numerous cancers (Table 1) and abundantly localized at the cell membrane surface and in late endocytic organelles (Lekishvili et al., 2008). TM4SF1 can interact with integrin-ß1 and integrin-α5 via its EC2 domain, and integrins-TM4SF1 protein complexes likely affect cell adhesion and motility (Hemler, 2005; Shih et al., 2009; Lee et al., 2011). The dynamics of TM4SF1 on the plasma membrane play an important role in cell motility. TM4SF1 location in the cytoplasm associated with the invasion of prostate cancer cells. In normal prostate epithelial cells, TM4SF1 expression could be detected in the apical membrane (Allioli et al., 2011). Inhibition of TM4SF1 expression was shown to affect prostate cancer cell motility, which might be related to the loss of surface expression in prostate cancer cells. In addition, TM4SF1 is recruited by and binds to TERMs through its intracellular domains, where it associates with other tetraspanins, such as CD63 and CD82, and thus influences the pro-migratory activity of cancer cells (Lekishvili et al., 2008). Downregulation of TM4SF1 expression on the cell surface led to significant increases in CD63 and CD82, which were negatively related to the motility of diverse cell types, overexpression of CD82 in prostate cancer cells resulted in alterations in the FAK-Lyn-p130CAS-CrkII signaling pathway, which correlated with decreased cell motility (Bernard, 2018). Ubiquitylation of TM4SF1 might prove to be an important factor in regulating the dynamics of CD63 on the plasma membrane. The subcellular expression of TM4SF1 and its expression level regulating by integrins and other tetraspanins can affect the motility of cancer cells.

**TABLE 1:** TM4SF1 expression in various cancers.

Cancer type	Techniques	TM4SF1 expression observations	References
Malignant melanoma	RT-PCR	Higher expression in melanoma cell lines than in melanocytes	Teutschbein et al., 2010
Prostate cancer (PCa)	qRT-PCR	Higher mRNA expression in PCa than in benign prostatic hyperplasia (BPH)	Allioli et al., 2011
Prostate cancer	qRT-PCR and WB	Overexpressed in PCa tissues and DU145 cells	Chen et al., 2019
Breast cancer	IHC	Highly expressed in >80% of breast cancer tissues when compared with adjacent normal tissue	Tu et al., 2012
Breast cancer	IHC	High expression in 137/209 breast cancer cases	Xing et al., 2017
Breast cancer	RT-PCR and WB	Significantly higher expression in BC tissues than in adjacent normal tissues	Fan et al., 2019
Pancreatic cancer	qRT-PCR	Higher mRNA expression in seven pancreatic cancer cell lines than in HPDE cell lines	Cao et al., 2015
Pancreatic cancer	IHC	Higher expression in cancer cells than in normal pancreatic tissues	Zheng B. et al., 2015
Pancreatic cancer	qRT-PCR and IHC	Highly expressed in pancreatic cancer tissues when compared with adjacent tissues	Cao et al., 2016
Pancreatic cancer	IHC and qRT-PCR	Upregulated in pancreatic cancer	Xu D. et al., 2020
Colorectal cancer (CRC)	qRT-PCR	Upregulated in CRC tissues and cell lines	Park et al., 2016
CRC	qRT-PCR and IHC	Upregulated in CRC tumor specimens when compared with adjacent normal tissues	Park et al., 2017
CRC	qRT-PCR and WB	Upregulated in human CRC tissues and cell lines	Park et al., 2018
Bladder cancer (BC)	qRT-PCR	Strongly upregulated in muscle invasive bladder cancer when compared with para-cancerous tissues and normal bladder tissues	Cao et al., 2018
Lung cancer	qRT-PCR and WB	Highly expressed in spheroid cells	Ma et al., 2018
Lung cancer	qRT-PCR	Upregulated in lung cancer cell lines and tissues	Ye et al., 2019
Lung cancer	qRT-PCR, WB, and IHC	Upregulated in lung cancer tissues and cell lines	Fu et al., 2020
Gastric cancer (GC)	IHC	Lower expression in gastric cancer tissues than gastric non-cancerous tissues	Peng et al., 2018
	qRT-PCR	Higher expression in glioma tissues	Wang et al., 2015
Ovarian cancer	IHC	Higher positive protein expression rate in epithelial ovarian cancer tissues (90.90%) than in benign ovarian tumor tissues (65.22%) and normal ovarian epithelial tissues	Gao et al., 2019
Thyroid cancer	oligonucleotide microarray analysis	Significantly upregulated in N1b papillary thyroid microcarcinoma	Lee et al., 2019
Liver cancer	qPCR	Elevated in hepatocellular carcinoma	Zhu C. et al., 2019

*RT-PCR, qPCR, and qRT-PCR: quantitative real-time polymerase chain reaction; WB: western blotting; IHC: immunohistochemistry.*

## TM4SF1 Promotes Cancer Proliferation and Migration

**FIGURE 2:**
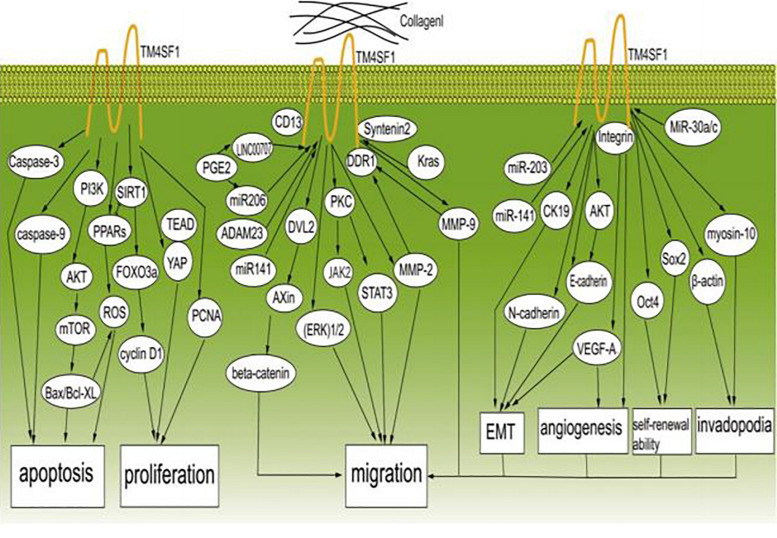
TM4SF1 promotes the development and migration of cancer cells. TM4SF1 promotes cancer by modulating various biological behaviors of cancer and inhibits cancer cell apoptosis by regulating caspase-9, caspase-3, BCL-2, Bax, the PI3K/AKT/mTOR pathway, and ROS-related pathways, and promotes cell proliferation by modulating the PCNA, cyclin D1, and YAP-TEAD pathways. TM4SF1 interacts with collagen I and induces metastatic reactivation. Soluble collagen I induces DDR1 aggregation at the cell surface and recruitment of TM4SF1 and enhances the interaction between DDR1 and TM4SF1, thus forming DDR1-TM4SF1 clusters at the cell surface. TM4SF1 functions as a membrane adaptor connecting DDR1 to syntenin2. Then, DDR1 activates PKC and the JAK2/STAT3 pathway through TM4SF1-mediated non-canonical DDR1 signaling by promoting the activation of PKC via recruitment of syntenin-2, thus inducing transphosphorylation and activation of JAK2/STAT3. TM4SF1 upregulates the expression of MMP-2 and MMP-9. TM4SF1 can form a complex with CD13 to promote cell migration and invasion. In addition, TM4SF1 activates the ERK 1/2 signaling pathway. TM4SF1 is a partner of DVL2 and positively modulates Wnt/beta-catenin signaling by enhancing DVL2-Axin interaction. TM4SF1 is induced by Kras signaling, and reduction of TM4SF1 promotes the ubiquitination of β-catenin. ADAM23 and miR-206 regulate the expression of TM4SF1, and prostaglandin E2 (PGE2) significantly inhibits the expression of miR-206, while increasing the expression of TM4SF1. LINC00707 functions as a molecular sponge of miR-206, and in this way indirectly regulates the expression of TM4SF1. miR-203 and miR-141 inhibit EMT by regulating TM4SF1-mediated modulation of E-cadherin, CK19, and VEGF-A expression. TM4SF1 regulates E-cadherin expression through the AKT signaling pathway. MiR-30a/c inhibits cancer stem-like characteristics by regulating TM4SF1. TM4SF1 suppresses the transcription of the pluripotency factors Sox2 and Oct4, suggesting that TM4SF1 promotes breast CSC traits. TM4SF1 interacts with integrins to stimulate VEGF-A to affect angiogenesis. TM4SF1 is associated with myosin-10 and β-actin, which are associated with filopodia generation. In conclusion, TM4SF1 promotes the migration of cancer cells by inducing EMT; enhancing self-renewal ability, tumor angiogenesis, and invadopodia formation; and regulating related signaling pathways.

Cancer metastasis is the major cause of death in cancer patients. Although the details of the underlying mechanisms are still not entirely clear, it is known that three main characteristics of cancer cells, i.e., constant proliferation, limited apoptosis, and migration, are important. Cancer cells avoid apoptosis by acquiring epigenetic modifications or genetic mutations in key regulators of apoptosis pathways. Numerous studies have shown that TM4SF1 plays an indispensable role in promoting cancer cell proliferation and migration through a series of signaling pathways (Figure 2).

Two studies reported that TM4SF1 was negatively correlated with apoptosis and positively associated with tumor growth in breast cancer (Sun et al., 2015; Fan et al., 2019). TM4SF1 was also positively correlated with cell migration, played a major role in metastatic reactivation, and promoted relapse in breast cancer (Simpson et al., 2010; Sun et al., 2015; Gao et al., 2016). In liver cancer cells, TM4SF1 promoted growth and inhibited apoptosis (Huang et al., 2016; Zhu C. et al., 2019); overexpression of TM4SF1 reduced apoptosis *in vitro* and increased proliferation *in vivo*, while decreased expression had the opposite effects (Huang et al., 2016). Downregulation of TM4SF1 impaired the ability of HCC to metastasize, whereas overexpression of TM4SF1 had the opposite effect (Huang et al., 2016; Zhu C. et al., 2019). In addition, TM4SF1 promoted the migration and invasion of lung cancer cells, and TM4SF1 expression regulated cell cycle arrest at the G2/M phase and promoted apoptosis in lung cancer cell lines A549 and H1299 cells (Chang et al., 2005; Ye et al., 2019). Linear regression analysis revealed that TM4SF1 expression levels increased with malignancy in five human lung cancer cell lines (CL1-0, CL1-1, CL1-2, CL1-3, and CL1-5) (Kao et al., 2003). Upregulated expression of TM4SF1 in lung cancer cells significantly increased their invasiveness *in vitro* and significantly decreased the survival time of xenograft mice, whereas silencing of TM4SF1 greatly inhibited tumor cell migration and invasion (Kao et al., 2003). Another study showed that decreased TM4SF1 expression enhanced the migration and invasion of pancreatic tumor cells *in vitro* (Zheng B. et al., 2015). In addition, silencing of TM4SF1 decreased the odds of lung and liver metastases in orthotopic pancreatic cancer (Cao et al., 2016). TM4SF1 also positively regulated the invasion and migration of human gastric cancer cells (Wei et al., 2018). TM4SF1 expression was higher in aggressively metastatic prostate cancer cells than in indolent, androgen-sensitive cancer cells (Chen et al., 2006). Overexpression of TM4SF1 significantly enhanced the invasion and migration of human prostate cancer cells (Chen et al., 2019) whereas silencing of TM4SF1 suppressed the migration and invasion of ovarian cancer cells (Gao et al., 2019). In addition, TM4SF1 expression was higher in metastatic cancer-derived tumors than in primary tumor-derived cells from a single colorectal cancer patient (Otsuka et al., 2001).

### Signaling Pathways Involving TM4SF1 That Promote Cancer Proliferation and Migration

Transmembrane 4 L six family 1 promotes migration and proliferation in multiple cancers through complicated signaling pathways containing diverse proteins that modulate the biological behaviors of cancer cells. One study showed that overexpression of TM4SF1 increased the expression levels of cyclin D1 and proliferating cell nuclear antigen (PCNA) and decreased the expression levels of caspase-9 and caspase-3 to inhibit apoptosis and promote proliferation (Huang et al., 2016; Cao et al., 2018). Cyclin D1 regulates the cell cycle at the G1 to S phase transition and promotes cell proliferation (Tao et al., 2016). High PCNA expression in liver cancer patients was related to increased proliferation and decreased post-operative disease-free survival time (Lim et al., 2020). Caspase-3 and caspase-9 are critical apoptotic proteases (Lossi et al., 2018). Overexpression of TM4SF1 significantly activated the phosphoinositide 3-kinase (PI3K)/protein kinase B (AKT)/mammalian target of rapamycin (mTOR) signaling pathway, which acts through the downstream apoptosis-related proteins B-cell lymphoma 2 (Bcl2), BCL-2 associated X (Bax), caspase-3, and caspase-9 to induce anti-apoptotic effects in breast cancer (Sun et al., 2015; Wei et al., 2018). Bcl2 and Bax are members of the Bcl2 family, which are key regulators of apoptosis (Wang et al., 2020). Bcl2 inhibits apoptosis by preventing cytochrome c release from the mitochondria into the cytoplasm. Bax is a pro-apoptotic protein that forms a heterodimer with Bcl2 to suppress its function (Wei et al., 2018). In human gastric cancer, TM4SF1 increased cell proliferation and inhibited apoptosis by increasing Bcl2 expression and decreasing Bax expression (Wei et al., 2018). Moreover, TM4SF1 promoted the proliferation and growth of human bladder cancer cells *in vitro* and *in vivo* and inhibited apoptosis by decreasing the ratio of Bax/B-cell lymphoma xl (Bcl-xl) (Cao et al., 2018). Bcl-xl inhibits apoptosis by protecting cells against reactive oxygen species (ROS) (Rodriguez-Gonzalez et al., 2018). MMP-2, MMP-9 and vascular endothelial growth factor (VEGF) are the downstream of TM4SF1, and TM4SF1 overexpression in turn upregulated the expression of MMP-2, MMP-9, and VEGF to stimulate endothelial cell division, proliferation and migration (Xue et al., 2017). In addition, reduced expression of β-catenin, VEGF, MMP-9, Snail, p-AKT, extracellular signal-regulated kinase (p-ERK), and vimentin and enhanced expression of E-cadherin could be inhibited by miR-206 via targeting TM4SF1 in PGE2-induced cells (Park et al., 2018). A study showed that the association between Axin and disheveled 2 (DVL2) was essential for breakdown of the β-catenin complex and activation of the Wnt/β-catenin cascade (Zhu C. et al., 2019). TM4SF1 was shown to be a partner of DVL2 that positively modulates Wnt/β-catenin signaling by enhancing DVL2-Axin interaction in HCC (Zhu C. et al., 2019). TM4SF1 expression was induced by Kras signaling, and reduction of TM4SF1 promoted the ubiquitination of β-catenin (Zhu C. et al., 2019). In addition to malignant tumors, Xu M. et al. confirmed that TM4SF1 overexpression in keloid promotes proliferation and cell motility by regulating the AKT and ERK1/2 pathways, which are related to cell apoptosis, proliferation, and motility (Xu M. et al., 2020).

Reactive oxygen species cause apoptosis in the surrounding microenvironment (Circu and Aw, 2010) and the reduction of TM4SF1 could induce cell cycle arrest and caused oxidative stress-induced apoptosis by increase ROS formation (Cao et al., 2018). Dysregulation of the cell cycle and ROS homeostasis in bladder cancer induced by silencing of TM4SF1 may involve the peroxisome proliferator-activated receptor (PPAR)-sirtuin1 (SIRT1) feedback loop, because crosstalk between SIRT1 and PPARγ plays a significant role in the modulation of apoptosis, antioxidant responses, and the cell cycle (Cao et al., 2018). In bladder cancer, TM4SF1 deficiency significantly upregulated PPARγ and forkhead transcription factor 3a (FOXO3a) and downregulated SIRT1 protein expression, forming a feedback loop that modulates the cell cycle (Cao et al., 2018). A previous study showed that PPARγ functions as a ligand-activated transcription factor that mainly regulates ROS homeostasis (Rocchi and Auwerx, 1999), and specific PPARγ ligands regulate the cell cycle both *in vitro* and *in vivo* (Theocharis et al., 2004). SIRT1 inhibits the cell cycle and is an important upstream target of FOXO3a that can downregulate cyclin D1 (Schmidt et al., 2002). These findings indicate that TM4SF1 can inhibit apoptosis through an ROS-related pathway. TM4SF1 was shown to enhance the expression of Yes-associated protein (YAP) and strengthened YAP-transcriptional enhancer activator domain (TEAD) interaction to regulate the progression of malignant tumor cells (Fu et al., 2020). YAP is an oncogene and a transcriptional activator of the Hippo pathway, and abnormal activation of this pathway can cause dysregulation of cell proliferation and apoptosis (Fu et al., 2020). Many studies have suggested that autophagy may also be associated with the progression of numerous cancers. Autophagy can inhibit tumorigenesis, and increasing TM4SF1 expression reduces the number of autophagosomes that could inhibit liver cancer cell autophagy, thus promoting tumorigenesis (Huang et al., 2016).

Numerous reports have demonstrated that ECM degradation is the first step leading to cancer invasion and subsequent metastasis (Gupta and Massague, 2006; Quail and Joyce, 2013). MMP-2 and MMP-9, which are the main members of the MMP family, are widely distributed and have the ability to destroy the basement barrier and promote the migration of capillary endothelial cells to initiate angiogenesis (Forsyth et al., 1999; Roomi et al., 2009; Buchheit et al., 2014). Upregulation of MMP-2 and MMP-9 expression induced by TM4SF1 increases ECM degradation, leading to the invasion and metastasis of pancreatic and liver cancer cells (Huang et al., 2016; Yang et al., 2017), and dysregulated expression of discoidin domain receptor 1 (DDR1) can inhibit the expression of MMP-2 and MMP-9 when TM4SF1 is silenced in pancreatic cancer (Cao et al., 2016; Yang et al., 2017). Gao et al. reported the expression of collagen I, DDR1, syntenin 2, TM4SF1, and signal transducer and activator of transcription-3 (P-STAT3) as well as their interaction in primary and metastatic breast carcinoma (Gao et al., 2016). They described a process in which, after destroying the surrounding basement membrane and making inroads into the interstitium, breast carcinoma cells come into contact with collagen I. Then, TM4SF1 interacts with collagen I and induces metastatic reactivation in several organs, including the lung, bone, and brain (Gao et al., 2016). Moreover, soluble collagen I not only induced DDR1 aggregation at the cell surface and concomitant recruitment of TM4SF1 but also enhanced the interaction between DDR1 and TM4SF1, forming large, persistent DDR1-TM4SF1 clusters at the cell surface (Gao et al., 2016). They also confirmed that the extracellular portion of DDR1 is indispensable for its interaction with TM4SF1 and that TM4SF1 functions as a membrane adapter connecting DDR1 with syntenin 2 (Gao et al., 2016). Finally, they confirmed that through TM4SF1-mediated non-canonical signaling, DDR1 could activate PKC and the Janus Kinase2 (JAK2)/STAT3 pathway by promoting activation of PKC via enlisting syntenin-2, thus inducing transphosphorylation and activation of JAK2/STAT3 (Gao et al., 2016). Further analysis revealed that non-canonical DDR1 signaling through STAT3 is activated during cancer growth and early metastasis (Gao et al., 2016). Based on these reports, we conclude that TM4SF1, syntenin-2, and DDR1 collaborate to recruit and activate PKCα and JAK2/STAT3.

The microRNAs miR-206 and miR-141 were shown to inhibit metastasis of triple-negative breast cancer and pancreatic cancer by negatively regulating *TM4SF1* by targeting its 3′-untranslated region (Xu et al., 2014; Park et al., 2018; Fan et al., 2019). Long intergenic non-coding RNA (LINC) 00707 was shown to act as a molecular sponge of miR-206 and indirectly regulate the expression of TM4SF1 (Zhu H. et al., 2019). Additionally, prostaglandin E2 (PGE2) significantly inhibited the expression of miR-206, thus increasing the expression of TM4SF1, and its induction promoted the migration of colorectal cancer cells (Park et al., 2018). In lung cancer, TM4SF1 significantly regulated PCNA and reduced the viability of lung cancer cells (Ye et al., 2019). Chang et al. showed that TM4SF1 can associate with CD13 and form a complex in lung cancer, prostate adenocarcinoma, and fibrosarcoma. The complex formed by TM4SF1 and CD13 allowed cells to migrate and invade (Chang et al., 2005). Knockdown of a disintegrin and metalloproteinase-23 (ADAM23) increased the expression of TM4SF1 in lung cancer (Ota et al., 2016). TM4SF1 could also significantly activate the extracellular regulated protein kinases extracellular signal-regulated kinase (ERK1/2) signaling pathway to increase the proliferation of DU145 cells and increase EMT in human prostate cancer (Chen et al., 2019).

### TM4SF1 Promotes EMT

Epithelial mesenchymal transition is a process in which cells lose their epithelial phenotype and gain a mesenchymal phenotype that is characterized by reduced expression of E-cadherin and cytokeratin19 (CK19) and increased expression of N-cadherin, fibronectin, and vimentin (Nieto et al., 2016; Santamaria et al., 2017). EMT is a major driver of cancer cell migration (Lee and Kong, 2016). One study showed that overexpression of TM4SF1 markedly increased EMT and enhanced the invasion and migration of human prostate cancer cells (Chen et al., 2019). Oral submucous fibrosis (OSF) is a potentially malignant tumor, and in OSF, miR-203 promotes EMT by targeting TM4SF1 and downregulating the expression of CK19 and E-cadherin while upregulating the expression of N-cadherin and vimentin (Zheng L. et al., 2015). In addition, TM4SF1 was shown to increase E-cadherin expression through the AKT signaling pathway in pancreatic and colorectal cancers (Zheng B. et al., 2015; Xu D. et al., 2020). Kim et al. (2018) demonstrated that miR-141 expression was related to TM4SF1 in pancreatic cancer, and upregulating miR-141, which decreased TM4SF1, reduced E-cadherin and VEGF-A to accelerate EMT in pancreatic cancer cells.

### TM4SF1 and Cancer Stem-Like Cells

Cancer stem cells are a small population of quiescent cells within cancer tissues that are characterized by their self-renewal ability and identified as a side population in primary cancers. They are localized within niches and have great potential to cause cancer growth and migration (Seo et al., 2007; Phay and Ringel, 2013; Xue et al., 2017; Najafi et al., 2019). TM4SF1 increased the carcinogenicity and self-renewal ability of esophageal CSCs to promote invasion and migration, suggesting that regulation of TM4SF1 is essential for controlling the self-renewal ability of esophageal CSCs (Xue et al., 2017). Bae et al. (2011) reported that TM4SF1 was abundantly expressed on mesenchymal stem cells (MSCs) and functions as a surface protein marker that distinguishes MSCs from diverse cell sources, particularly fibroblastic connective tissues. MiR-30a/c significantly decreased the cancer stem-like characteristics of non-small cell lung cancer-initiating cells by inhibiting *TM4SF1 in vitro* and *in vivo* (Ma et al., 2018). Silencing of TM4SF1 completely suppressed the transcription of the pluripotency factors sex-determining region Y box 2 (Sox2) and octamer-binding transcription factor-4 (Oct4), suggesting that TM4SF1 could promote the CSC traits of breast cancer (Gao et al., 2016). These findings suggest that TM4SF1 could be useful as a CSC marker.

### TM4SF1 and Tumor Angiogenesis

Tumor angiogenesis is required for the progression and migration of malignant tumors (Lugano et al., 2019). Shih et al. (2009) showed that TM4SF1 was highly expressed in the blood vessels of multiple malignant tumors and many types of cultured endothelial cells (ECs) derived from various tissues, including the coronary artery, umbilical vein [human umbilical vein endothelial cells (HUVECs)], pulmonary artery, dermal microvasculature, and the lymphatic system, and was necessary for maintaining their biological functions. After these *in vitro* experiments, they showed that knock down of TM4SF1 *in vivo* influenced vascular angiogenesis in the Ad-VEGF-A164 mouse ear angiogenesis model (Shih et al., 2009). Furthermore, they also found that TM4SF1 could interact with integrin subunits AV, B3, and B5 to promote angiogenesis and mediate endothelial cell migration only after VEGF-A or thrombin stimulation. In summary, TM4SF1 is a key modulator of EC functions *in vitro* and cancer angiogenesis *in vivo* (Shih et al., 2009).

### TM4SF1 and Invadopodia

Invadopodia have emerged as key cellular structures that modulate the metastasis and invasion of numerous cancers, and various proteins regulate invadopodia formation and function (Jacob and Prekeris, 2015). Yang et al. (2017) found that the expression of tumor-associated TM4SF1 could affect the formation and function of invadopodia in pancreatic cancer. In addition, it was shown that TM4SF1 promoted the extension of filopodia, and TM4SF1-knockdown HUVECs became stationary, senescent, and failed to generate filopodia (Shih et al., 2009). TM4SF1 was found to be located in a regularly spaced, banded pattern of TM4FS1-enriched domains that were essential for ECs to form “nanopodia,” which have poor F-actin and are especially long (up to 50 μm) and thin (100–300 nm wide) (Zukauskas et al., 2011). ECs form nanopodia when they migrate and interact with nearby cells. When TM4SF1 mRNA levels increased, ECs formed more and longer nanopodia around the circumference of the cells (Zukauskas et al., 2011). Forced overexpression of TM4SF1 in fibroblasts at levels similar to those in ECs led to the generation of TM4SF1-banded nanopodia and broadened, EC-like lamellipodia (Zukauskas et al., 2011). Further studies also showed that TM4SF1 is associated with myosin-10 and β-actin, which are involved in filopodia generation and cell migration (Zukauskas et al., 2011). These data indicate that TM4SF1 is important for generating invadopodia by cancer cells.

## Clinical Prognosis of TM4SF1 Expression

As shown in Table 2, shorter median survival time, shorter median overall survival, shorter progression-free survival, early post-operative relapse, older age, and smoking were associated with high TM4SF1 expression, which has prognostic value, is a risk factor for poor outcomes, and could be useful as a tumor biomarker for patients with lung cancer (Kao et al., 2003; Ma et al., 2018; Ye et al., 2019). In human glioma, the expression level of TM4SF1 was markedly higher in higher-grades (III–IV) glioma tissues than in lower grade (I–II) tumor tissues (Wang et al., 2015). TM4SF1 expression and world health organization (WHO) grades (WHO grades for glioma patients are the gold standard for determining prognosis and the tumors are graded into four malignancy grades. Grade I is the least and grade IV is the most malignant tumors) were independent prognostic factors influencing survival in glioma. Lower pathological grade, macroscopic total resection, younger age, higher Karnofsky performance score, and lower TM4SF1 expression were associated with longer overall survival of patient with glioma. These findings show that TM4SF1 could be a useful prognostic marker for glioma patients (Wang et al., 2015). Xu D. et al. (2020) showed that elevated expression levels of TM4SF1 and advanced clinicopathological features are associated with poor prognosis in pancreatic cancer patients. TM4SF1 expression was related to tobacco smoking, tumor size, diabetes, clinical stage, T stage, lymph node metastasis, tumor cell venous invasion, distant tumor metastasis, lymphatic invasion, and poor overall survival in patients with pancreatic cancer (Xu D. et al., 2020).

**TABLE 2:** The relationships between TM4SF1 and clinical prognosis.

Cancer type	Techniques	Notes	References
Malignant pleural mesothelioma (MPM)	qRT-PCR	TM4SF1/PKM2 and TM4SF1/ARHGDIA gene expression ratios could predict survival	Gordon et al., 2009
Ovarian cancer	IHC	TM4SF1 protein expression was correlated with FIGO stage and histological grade	Gao et al., 2019
Glioma	Kaplan–Meier analysis with the log-rank test, WB, and IHC	TM4SF1 expression increased with tumor grade, and high expression had a significant negative impact on overall survival	Wang et al., 2015
Colorectal cancer	IHC and qRT-PCR	TM4SF1 expression was significantly associated with advanced stage and lymph node status compared with adjacent normal tissues	Park et al., 2017
Hepatocellular carcinoma	A linear association test	TM4SF1 was an indicator of poorly differentiated HCC at initial diagnosis	Shao et al., 2017
Breast cancer	IHC	High TM4SF1 expression was associated with advanced histological grade, negative estrogen receptor, progesterone receptor status, and shorter disease-free survival and overall survival	Xing et al., 2017
Bladder cancer	IHC	High TM4SF1 expression was associated with T stage, TNM stage, lymph node metastasis status, and survival rate	Cao et al., 2018
Gastric cancer	IHC	TM4SF1 expression was correlated with depth of invasion, nodal metastasis, TNM stage, grade differentiation, Lauren classification, 5-year survival rate, and median survival time	Peng et al., 2018
Papillary thyroid microcarcinoma	qRT-PCR	TM4SF1 expression was higher in N1b than in N0 PTMCs	Lee et al., 2019
Lung cancer	UALCAN database (www.ualcan.path.uab.edu/)	High TM4SF1 expression was associated with older patient age, smoking habits, and poor survival	Ye et al., 2019
Lung cancer	Kaplan-Meier analysis	TM4SF1 expression was correlated with survival time, tumor size, lymph node metastasis, distant metastasis, overall survival, and clinical stage	Fu et al., 2020
Lung cancer	qRT-PCR	Increased TM4SF1 expression was significantly associated with early post-operative relapse and shorter survival	Kao et al., 2003
Lung cancer	Kaplan-Meier survival analysis	High TM4SF1 expression was correlated with shorter median overall survival and PFS	Ma et al., 2018
Pancreatic cancer	published profiles of the TCGA dataset	TM4SF1 expression was associated with tobacco smoking, diabetes, tumor size, clinical stage, T stage, lymph node metastasis, distant tumor metastasis, tumor cell venous invasion, lymphatic invasion, and poor overall survival	Xu D. et al., 2020
Pancreatic cancer	IHC	High TM4SF1 expression group showed better survival and lower locoregional recurrence rate than the low expression group	Zheng B. et al., 2015

Transmembrane 4 L six family 1 expression was positively correlated with tumor grade and could help HCC distinguish HCC with grade 3 from grade 1. However, it was not associated with the overall survival of patients with HCC (Shao et al., 2017). TM4SF1 expression was upregulated in colorectal cancer specimens and was significantly related to advanced stage and lymph node status when compared with para-cancerous tissues (Park et al., 2017). Furthermore, TM4SF1 was a more sensitive and precise marker for the diagnosis and detection of colorectal cancer than the carcinoembryonic antigen (Schiedeck et al., 2001). High TM4SF1 expression was significantly associated with advanced histological grade, estrogen receptor negativity, and progesterone receptor status in primary tumors and with reduced relapse-free survival in estrogen receptor breast cancer patients when compared with ER+ patients, and this may be the reason that ER signaling inhibits TM4SF1 expression in breast cancer (Simpson et al., 2010). Triple-negative breast cancer has a tendency toward higher TM4SF1 protein levels than other types of breast cancer (Xing et al., 2017). Patients with breast cancer with higher TM4SF1 protein levels have shorter disease-free survival and overall survival time (Xing et al., 2017). Cao et al. (2018) reported that high-grade papillary breast cancer and infiltrating breast cancer have higher TM4SF1 expression levels than low-grade papillary breast cancer and para-cancerous breast cancer tissues. They also found that TM4SF1 expression was positively correlated with T stage, TNM stage (the size of tumor, lymph node metastasis, and distant metastasis), and lymph node metastasis status and negatively correlated with cumulative survival, overall survival, and disease-free survival time (Cao et al., 2018). However, other clinical parameters, including sex, tumor grade, age, and tumor size, were not correlated with TM4SF1 (Cao et al., 2018). Epithelial ovarian malignant tumor tissues and low differentiation tissues or tissues from International Federation of Gynecology and Obstetrics (FIGO) stages III–IV patients had higher TM4SF1 expression levels than para-cancerous tissues, normal ovarian epithelial tissues, tumor tissues with high differentiation, and I–II FIGO stage tumors (Gao et al., 2019). Both the histological stage and FIGO grade affected the prognosis of ovarian cancer patients; thus, TM4SF1 expression was not an independent factor influencing the prognosis of ovarian cancer patients (Gao et al., 2019). Allioli et al. (2011) showed that TM4SF1 expression was significantly higher in N1b papillary thyroid microcarcinomas (PTMCs) than in N0 PTMCs.

Transmembrane 4 L six family 1is a tumor-associated protein that is widely expressed in many kinds of human carcinomas. However, the exact role of TM4SF1 in cancer remains controversial. Results of Peng et al. (2018) showed that TM4SF1 was a tumor suppressor for GC and might function as a tumor inhibitor in GC as well as a novel prognostic marker. Low expression of TM4SF1 is associated with carcinogenesis and development, tumor progression and invasion of gastric cancer, and poor overall survival of patients with GC. Higher level expression of TM4SF1 was involved in GC tissues of higher grade differentiation. In addition, Zheng B. et al. (2015) reported that patients with high levels of TM4SF1 showed longer survival time than those with low TM4SF1 levels. *In vitro*, reduced TM4SF1 expression after TGF-ß1-induced EMT enhanced the migration and invasion of pancreatic cancer cells partially via decreased E-cadherin expression. Results of Abba et al. (2004) showed that the expression of TM4SF1 was downregulated in invasive breast carcinomas and upregulated in normal breast tissue by serial analysis of gene expression (SAGE) of various normal and tumor libraries. Moreover, Studies have shown that TM4SF1 could serve as a predictor of post-surgical treatment efficacy in patients with malignant pleural mesothelioma (MPM), and patients with higher TM4SF1 expression levels have relatively good prognosis (Gordon et al., 2003, 2009, 2011).

The function of TM4SF1 in different kinds of cancer was shown in Table 2. Different literatures reported different clinical prognostic significance about the expression of TM4SF1. High expression of TM4SF1 predicted a poor prognosis in patients with glioma, colorectal cancer, breast cancer, papillary thyroid carcinomas and ovarian cancer but predicted good prognosis in GC, pancreatic cancer, breast cancer and MPM. The reason may be that TM4SF1 is regulated by different signal pathways and different subcellular location of TM4SF1 plays different roles in the development and progression of different malignant tumors, which associated with different clinical prognosis.

## TM4SF1 and Drug Resistance

Drug resistance is a major cause of treatment failure in cancer patients receiving cytotoxic or targeted therapies for disseminated cancer (Dannenberg and Berns, 2010), and numerous reports have shown that TM4SF1 is positively associated with drug resistance. Higuchi et al. (2003) demonstrated that upregulation of TM4SF1 expression in a head and neck squamous cell carcinoma cell line is related to *cis*-diammine dichloro platinum (cDDP) resistance, and the mechanism of resistance may involve TM4SF1-mediated regulation of cell surface molecules and related signaling proteins associated with cDDP cell entry. Moreover, TM4SF1 increased chemoresistance to gemcitabine by increasing the expression of multidrug resistance genes, such as ATP-binding cassette transporter B1 (*ABCB1*) and ATP-binding cassette C member 1 (*ABCC1*), which are involved in chemoresistance in pancreatic cancer cells (Cao et al., 2015). In MCF-7^+^p23 breast cancer cells, TM4SF1 was markedly increased and ABCC3 was obviously increased, and upregulation of ABCC3 in these cells promoted doxorubicin resistance (Simpson et al., 2010). Long-term estradiol deprivation caused a change in TM4SF1 expression levels, resulting in hormonal failure in breast cancer (Santen et al., 2005). The mechanisms underlying TM4SF1-related paclitaxel and cisplatin resistance in lung cancer include tumor resistance to apoptosis, cell cycle regulation, interaction with DDR1, and regulating key genes in both the mitogen-activated protein kinase and ERK/Akt-mTOR pathways (Ye et al., 2019). In addition, TM4SF1 increased cisplatin resistance in esophageal CSCs (Xue et al., 2017). Based on these results, TM4SF1 might be a promising target for overcoming drug resistance in multiple cancers.

## TM4SF1 and Immunotherapy

Immunotherapy has proven to be an effective therapeutic approach in a variety of cancers. Treatment with bicalutamide or 5-alpha-reductase downregulated the expression of TM4SF1 in prostate cancer cells, suggesting that TM4SF1 could be used to guide clinical treatment (Buhler et al., 2010). As TM4SF1 expression is higher on the surface of malignant tumors and is significantly associated with cancer development and metastasis, it is an alluring target to antibody-based immunotherapy. mAb-L6 is a monoclonal antibody which binds to an integral membrane glycoprotein that is highly expressed in multiple carcinomas, which corresponds to TM4SF1. This antibody induces complement-dependent cytotoxicity with human complement and antibody-dependent cellular cytotoxicity with human mononuclear cells (Hellstrom et al., 1986a; Kao et al., 2003). Due to the clustering of TM4SF1 on the cell surface, this antibody could kill TM4SF1-positive cancer cells and inhibit cancer cell invasion (Hellstrom et al., 1986a; Kao et al., 2003).

In a phase I trial of mAb-L6, 19 patients with advanced breast, colon, ovarian, and lung cancers that highly expressed TM4SF1 were selected, and 18 patients were evaluable. The only side effects were headaches and fever at the highest dose (Goodman et al., 1990). A similar study was conducted using a chimeric (mouse-human) monoclonal antibody against L6-Ag (chL6) in lung, colon, and breast cancer patients, which showed a curative effect with side effects (fever, chills, and nausea) lasting 1–2 days (Goodman et al., 1993). Two subsequent clinical trials showed that chL6 was an appropriate antibody for delivering antitumor agents due to its low immunogenicity and satisfactory tumor binding specificity (Goodman et al., 1993). Experiments conducted in mice showed that the chL6 antibody caused antibody-dependent cellular cytotoxicity at a significantly lower concentration than that required for the mouse and it was even effective against melanoma expressing low levels of TM4SF1 (Liu et al., 1987). Induction of T-cell immune reactions against TM4SF1 may be a meaningful therapeutic approach for diverse cancers. Tu et al. (2012) reported that peptide 5 immunization could induce HLA-A2–restricted TM4SF1-specific antitumor immunity. Peptide 5 is a TM4SF1 CTL (cytotoxic CD8 T-cell) epitope and may induce T-cell reactions after DNA immunization and increase peptide-specific cytotoxic T cells (CD107a+, CD8+), thus inhibiting the growth of EL4/TM4SF1/HLA-A2 tumor cells in A2 Tg mice.

Due to their safety and flexibility, synthetic peptides have become attractive molecules for cancer immunotherapy. The EP1 peptide is a linear B-cell epitope of TM4SF1 that can induce anti-tumor activity against malignant tumor cells that express TM4SF1 (Lin et al., 2016). The B cell epitope was incorporated with a cytotoxic T lymphocyte and a helper T epitope to form a chimeric long peptide that has strong cellular and humoral immunity and inhibits tumor migration (Lin et al., 2016). TM4SF1 is expressed not only in numerous cancers but also in tumor vessels and is associated with angiogenesis. Lin et al. (2014) established a model using engineered human vessels in immunodeficient nude mice by generating a human vascular network in Matrigel. An anti-TM4SF1 EC2 mAb called 8G4 was able to kill human prostate cancer cells in the Matrigel by inducing human MSCs and human endothelial colony-forming cells to destroy the human vascular components (Lin et al., 2014). TM4SF1 has potential as a dual therapeutic target by using an antibody drug conjugate (ADC) because of its prominent surface location on tumor cells and tumor-associated endothelium. 8G4 (a mouse anti-human TM4SF1 monoclonal antibody) was efficiently taken up by cultured ECs in a dynamin-dependent and clathrin-independent manner and transported through the cytoplasm along microtubules and across nuclear pores into the nucleus (Sciuto et al., 2015).

The results of one study suggested that the binding of two novel mAb-drug conjugates significantly inhibited both tumor xenografts and the tumor vasculature. A conjugate consisting of 2A7A, a humanized TM4SF1 mAb, and the auristatin cytotoxic agent LP2 (chemical name mc-3377) damaged the ECs in the mouse tumor vasculature and inhibited the growth of several tumor xenografts with dosage-related toxicity (Visintin et al., 2015). They also reported that combining two types therapies (v1.10-LP2 and 2A7A-LP2) was more effective than treatment with the ADC alone in nude mice with colon, pancreas, prostate, and lung cancers (Visintin et al., 2015). Sher et al. (2019) designed an endoplasmic reticulum-targeting sequence (adenovirus E3/19K protein) and placed it at the N-terminus of TM4SF1 to facilitate major histocompatibility complex class I antigen presentation to CD8^+^T cells to enhance the efficacy of immunotherapy. Transfection with a plasmid containing TM4SF1 fused with the ER-targeting sequence (pEKL6) induced higher levels of TAL6 antigens in the ER than transfection with full-length TM4SF1 (pL6) in mammalian cells (Sher et al., 2019). pEKL6 not only induced stronger TM4SF1-specific CTL responses but also higher antibody titers after intramuscular (IM) immunization via electroporation in HLA-A2 transgenic mice (Sher et al., 2019). In addition, higher levels of protective antitumor immunity to inhibit tumor growth were induced by immunization with pEKL6 than by immunization with pL6 in animal models of thymoma and melanoma (Sher et al., 2019). Furthermore, pEKL6 had long-term antitumor immunity effects against cancer recurrence, and further study showed that CD4^+^T, CD8^+^T, and natural killer cells play important roles in the effector mechanisms of pEKL6 immunization (Sher et al., 2019).

## Roles of Other TM4SFs in Cancer Development and Progression

The tetraspanin superfamily includes TM4SF1, TM4SF4, TM4SF5, TM4SF18, TM4SF19, and TM4SF20 (Zhang et al., 2001). It is reported that TM4SF4 and TM4SF5 could promote hepatocellular carcinoma (HCC) cell growth and metastasis both *in vivo* and *in vitro* (Lee et al., 2008; Li et al., 2012). Choi et al. (2017) demonstrated that TM4SF4 was involved in epithelial mesenchymal transition (EMT) and cancer stem cell (CSC) properties of non-small cell lung cancer (NSCLC) through the regulation of osteopontin. TM4SF4 triggered OPN expression by creating a positive feedback autocrine loop with JAK2/STAT3 or focal adhesion kinase (FAK)/STAT3 pathways. Blocking the activity of TM4SF4 using specific antibody and short interfering RNA (siRNA) can be a promising therapeutics against TM4SF4-overexpressing cancer (Wang et al., 2013; Choi et al., 2014).

By using chimeric constructs, Jin-Gyu Cheong et al. reported that the C-terminus of TM4SF5 played an important role to regulate the diverse metastatic functions, and could positively replace the C-termini of TM4SF1 or TM4SF4, which increased the ability of cancer cell proliferation migration, and invasion (Cheong et al., 2017). However, the cytoplasmic or extracellular regions of TM4SF1 substituted by corresponding sequences of TM4SF5 did not prevent localisation of the chimeric proteins to Lamp2-positive organelles (Lekishvili et al., 2008). TM4SF5 was highly expressed in liver (Lee et al., 2008) and prostate cancers (Muller-Pillasch et al., 1998) and interacted with integrins α2, α5, and β1, EGFR, IL6R, CD151, FAK, and c-Src (Lee, 2014). TM4SF5 interacted with integrins α2, α5, or β1 and played roles in cell adhesion and migration through actin reorganization (Lee, 2014). TM4SF5 could interact with the cytoplasmic tail of integrin α5 and this interaction with integrin α2β1 relies on the N-glycosylation status in the LEL (long extracellular loop 2) or structural intactness of the LEL of TM4SF5 (Lee et al., 2011). Interference of the association between TM4SF5 and integrin α2 using anti-TM4SF5 reagent causes recoveries in spreading on and migration toward collagen I (Lee et al., 2011).

TM4SF5 could cooperate with integrin α5 to transduce signaling through FAK-c-Src complex activation and STAT3 phosphorylation to induce the expression of VEGF in cancerous epithelial cells (Choi et al., 2009). TM4SF5 promoted self-renewal and circulating tumor cell (CTC) properties, and mediated metastasis through the TM4SF5/CD44/c-Src/STAT3/Twist1/Bmi1 pathway (Lee et al., 2015). In addition, a study demonstrated that TM4SF5 could through form a complex with mTOR and SLC38A9 on lysosomal membranes in an arginine-regulated manner, lead to mTOR/S6K1 activation (Jung et al., 2019). Epidermal growth factor receptor (EGFR) signaling pathway is involved in the expression of TM4SF5 (Kang et al., 2012). The interaction between TM4SF5 and EGFR is positively correlated with the gefitinib resistance even without EGFR mutation following TM4SF5 overexpression (Lee et al., 2012). In addition to integrins, TM4SF5 can also interact with FAK and active FAK by releasing the inhibitory intermolecular interaction. TM4SF5 recruits with different actin-organizing molecules including neuronal Wiskotte, Aldrich Syndrome protein (N-WASP), actin-related protein 2 (Arp2) in invadopodia to promote migration/invasion of HCC (Lee et al., 2011; Jung et al., 2012; Lee, 2015). Furthermore, TM4SF5 was capable to decrease expression of E-cadherin, zonula occludens 1 (ZO1) and increase expression of α-smooth muscle actin (α-SMA), which decreased the adhesion of cells and enhanced the ability to invasion (Lee et al., 2008; Lee and Kong, 2016). TM4SF5 promoted CDK4 and cyclin D1 into nucleus and facilitated G1/S phase progression, which could be blocked by p27Kip1 siRNA or by infection of active RhoA (Kim et al., 2010). Similar to the tetraspanins, TM4SF5 can localize at its own TEM. CD151 is increased in TM4SF5-positive cells whereas CD9, CD63, CD82, CD105, and CD117 are not (Kang et al., 2014). TM4SF5 is upstream of CD151 and can co-localize with CD151 on the plasma membrane, which regulated the levels of CD151 mRNA and protein (Lee, 2014). The expression TM4SF5 could cause internalization of CD63, which resulted in the decrease of CD63 level at the membrane surface and disrupted its tumor-suppressing action (Kang et al., 2014). CD63 was the first characterized tetraspanin, which abundantly expressed in the early stage in melanoma, however, its level decreases in the process of malignant progression, so CD63 surface levels is negatively correlation with invasiveness (Pols and Klumperman, 2009).

Singhal et al. (2019) investigated the expression and localization of the TM4SF18 protein in normal human pancreas and pancreatic ductal adenocarcinoma tissue and demonstrated that TM4SF18 is highly expressed in pancreatic ductal adenocarcinoma epithelium and weakly expressed in normal ducts. TM4SF18 could induce the expression of VEGF and amplify VEGF signaling to speed angiogenesis (Page et al., 2019). TM4SF20 is another member of tetraspanin superfamily. The interactions with TM4SF4 mediated by this surface during translocation may be critical for regulated alternative translocation of TM4SF20 (Wang et al., 2019).

## Conclusion

Since its discovery, TM4SF1 has received much attention due to its ability to regulate a variety of cellular biological functions in malignant tumors, including motility, proliferation, and apoptosis and its association with the clinical prognosis of cancer patients. Interactions between TM4SF1 and its receptors can activate numerous intracellular pathways that control the progression and metastasis of many malignancies. In nearly all cancers described in this review, increased expression of TM4SF1 promoted the development of cancer and cancer cell migration by inducing EMT, angiogenesis, invadopodia formation, and self-renewal ability. The close relationship between TM4SF1 and clinical prognosis makes it a valid biomarker for cancer diagnosis, and as TM4SF1 may promote drug resistance in cancer, it is also a potential therapeutic target.

## Author Contributions

SZ and XY designed the study, contributed to manuscript writing, and approved the manuscript before submission. FF, MZ, and QZ collected the literatures and approved the manuscript before submission. KZ, ZL, and HZ gave constructive comments on the manuscript, and approved the manuscript before submission. All authors contributed to the article and approved the submitted version.

## Conflict of Interest

The authors declare that the research was conducted in the absence of any commercial or financial relationships that could be construed as a potential conflict of interest.

## Abbreviations

α -SMA: α -smooth muscle actin
ABCB1: ATP-binding cassette transporter B1
ABCC1: ATP-binding cassette C member 1
ADAM23: a disintegrin and metalloproteinase
ADC: antibody drug conjugate
ADCC: antibody-dependent cellular cytotoxicity
AKT: protein kinase B
Bax: BCL-2 associated x
BC: bladder cancer
BCa: Breast cancer
Bcl2: B-cell lymphoma 2
CCG: Cys-Cys-Gly
CDC: complement dependent cytotoxicity
cDDP: *cis*-diammine dichloro platinum
chL6: chimeric monoclonal antibodyL6
CTL: cytotoxic CD8 T cell
CK19: cytokeratin 19
CRC: colorectal cancer
CSCs: cancer stem cells
CTC: circulating tumor cell
DDR1: discoidin domain receptor 1
DVL2: dishevelled 2
EC: endothelial cells
EC2: extracellular loop 2
ECFC: endothelial colony-forming cells
EMT: epithelial mesenchymal transition
ERK: extracellular signal-regulated kinase
FAK: focal adhesion kinase
FIGO: International Federation of Gynecology and Obstetrics
FOXO3a: forkhead transcription factor 3a
YAP: Yes-associated protein
GC: gastric cancer
HCC: hepatocellular carcinoma
HNSCC: head and neck squamous cell carcinoma
IHC: immunohistochemical
JAK2: Janus Kinase2
LINC00707: long intergenic non-coding RNA 00707
mAb: monoclonal antibody
MDR: multidrug resistance
MHC: major histocompatibility complex
MMP-2: matrix metalloproteinase
MPM: malignant pleural mesothelioma
MSC: mesenchymal stem cells
MST: shorter median survival time
mTOR: mammalian target of rapamycin
NSCLC: non-small cell lung cancer
N-WASP: neuronal Wiskotte Aldrich Syndrome protein
Arp2: actin-related protein 2
ZO1: zonula occludens 1
Oct4: octamer binding transcription factor-4
OSF: Oral submucous fibrosis
PC: Prostate cancer
PCNA: proliferating cell nuclear antigen
pEK: ER-targeting sequence
PFS: progression-free survival
PGE2: prostaglandin E2
PI3K: phosphoinositide 3-kinase
PKC: protein kinase C
PPARs: peroxisome proliferator-activated receptors
PXSC: Phe-X-Ser-Cys
EGC: Glu-Gly-Cys
ECM: extracellular matrix
qRT-PCR: Quantitative Real-time Polymerase Chain Reaction
SIRT1: sirtuin1
SITAC: syntenin-2
Sox2: sex-determining region Y box 2
SP: side population
STAT3: signal transducer and activator of transcription-3
TEAD: transcriptional enhancer activator domain
TM4SF1: Transmembrane 4 L six family 1
TNBC: triple-negative breast cancer
UTR: untranslated region
VEGF: vascular endothelial growth factor
WB: western blot.
